# A Simple and Scalable Chopped-Thallus Transformation Method for *Marchantia polymorpha*

**DOI:** 10.3390/plants14040582

**Published:** 2025-02-14

**Authors:** Rituraj Batth, Andisheh Poormassalehgoo, Kritika Bhardwaj, Elżbieta Kaniecka, Shino Goto-Yamada

**Affiliations:** 1 Malopolska Centre of Biotechnology, Jagiellonian University, 30-387 Krakow, Poland; 2 Doctoral School of Exact and Natural Sciences, Jagiellonian University, 30-387 Krakow, Poland

**Keywords:** *Marchantia polymorpha*, liverwort, *Agrobacterium*-mediated transformation, transgenic, chopping, regeneration, thallus, genetic modification

## Abstract

The liverwort *Marchantia polymorpha* has emerged as a valuable model for studying fundamental biological processes and the evolutionary history of land plants. *Agrobacterium*-mediated transformation is widely used for genetic modification of *M. polymorpha* using spores, thalli, and gemmae. While spores offer high transformation efficiency, they result in diverse genetic backgrounds due to sexual reproduction. Conversely, thallus- and gemma-based methods maintain genetic consistency but are impractical for large-scale applications. To address these limitations, we developed a novel chopped-thallus transformation method. This technique improves transformation efficiency by generating numerous thallus fragments through chopping and optimizing the regeneration duration. The method demonstrated superior transformation efficiency compared to traditional approaches and achieved sufficient numbers of transformants using simplified Gamborg’s B5 medium, previously considered suboptimal. This scalable and straightforward method enables the generation of large numbers of genetically consistent transformants, facilitating high-throughput experiments, including mutant screening and other large-scale applications.

## 1. Introduction

The liverwort *Marchantia polymorpha* (hereafter Marchantia) belongs to one of the earliest diverging lineages of land plants, along with hornworts and mosses [1,2]. Its genome has been fully sequenced, and the data are publicly available. Marchantia’s simple morphology and compact structure make it particularly suitable for laboratory studies [1,3,4]. In addition to sexual reproduction, Marchantia can propagate asexually through detached thalli or gemmae, which is beneficial for analyzing mutants with reproductive abnormalities. Moreover, its haploid-dominant life cycle and low level of gene redundancy have facilitated its use in genetic studies [1,3,4]. For these reasons, Marchantia has emerged as a key model organism in plant research.

Genetic modification is an essential technique for studying molecular biology, with significant implications for agriculture, biotechnology, and fundamental biological research. Several methods have been proposed for transforming Marchantia, including *Agrobacterium*-mediated stable and transient transformations [5,6,7,8] and particle bombardment [9]. *Agrobacterium*-mediated transformation is a widely used approach due to its efficiency in stably integrating foreign DNA into plant genomes. In Marchantia, *Agrobacterium*-mediated transformations have been established using spore-derived tissues, thalli, and gemmae to obtain stable transformants. Thallus-based transformations involve excising apical meristems, regenerating fragments on plates, and co-cultivating them with *Agrobacterium* in immersion culture [6]. The spore-based method is the most efficient to date, where spores germinate into immature thalli, which are then co-cultivated with *Agrobacterium* [5]. The AgarTrap method, which combines plant culture, infection, and selection on a single plate, has also been developed [7,10].

Although *Agrobacterium*-mediated transformation is widely used in Marchantia species, several challenges remain unresolved. First, transformation efficiency does not always reach the high levels reported in the literature. For example, while simple constructs often result in high efficiency, transformation efficiency can drop significantly depending on the construct. Therefore, improving the overall efficiency, even at a standard level, could also enhance the success rate of more challenging constructs. Second, traditional transformation methods often rely on complex media compositions [5,6]. Therefore, evaluating the efficiency of simpler, more accessible media would be highly beneficial. Moreover, obtaining a large number of transformants, such as those used for mutant screening, is crucial for large-scale experiments. The most efficient spore-based method presents challenges: spores generated from the mating of male and female strains can lead to genetically heterogeneous populations. While the male Takaragaike-1 (Tak-1) and female Tak-2 strains share highly similar traits, they differ in reproductive chromosomes, numerous SNPs, and subtle morphological variations [1,5,11]. Recent studies have also reported differences in stress responses between male and female plants [12]. Although the Tak-1 inbred female line BC3-38 has been established from an F1 hybrid of Tak-1 and Tak-2 [5,13], it suffers from low fertilization success. Although spore-based transformation remains the most efficient method, the use of asexually propagated thalli is preferred due to these genetic considerations. Additionally, mutants with reproductive defects, such as autophagy mutants [14], are unable to produce spores from their genetic background. These limitations highlight the need for a simplified, efficient transformation method utilizing vegetatively propagated tissues, such as regenerating thalli.

Marchantia, like other bryophytes, has a high regenerative ability, allowing for the easy establishment of new plants [15,16]. Removing the meristem can induce the regeneration of thallus edges, leading to the formation of new tissues within five days [6]. Protoplast cells in Marchantia can also regenerate their cell walls and reproduce a whole plant [17,18], suggesting the potential for regenerating entire plants from single cells. In traditional thallus transformation methods, one Marchantia plant produces four thallus fragments for subsequent transformation [6]; however, fragments can potentially be smaller, with studies reporting transformations using fragments as small as 4–5 mm square [19]. In the amphibious liverwort *Riccia fluitans* and the hornwort *Anthoceros agrestis*, *Agrobacterium*-mediated transformation has been performed with fragmented thalli obtained by chopping [20,21,22].

As previously mentioned, several challenges persist, including inconsistent transformation efficiencies, the reliance on complex media compositions, and difficulties in producing large numbers of genetically consistent transformants. To address these issues, we developed an optimized *Agrobacterium*-mediated transformation protocol using finely chopped thallus fragments, which takes advantage of the remarkable regenerative capacity of Marchantia. We developed a scalable method that generates numerous transformants from minimal starting material. We evaluated factors influencing transformation efficiency, such as regeneration duration and medium composition. This method offers a solution for the genetic modification of Marchantia, enabling large-scale applications, including mutant screening and other high-throughput studies.

## 2. Materials and Methods

### 2.1. Plant Material and Preparation

The male accession of *Marchantia polymorpha* Takaragaike-1 (Tak-1) [5,23] was used in this study. Plants cultured on a solid ½ Gamborg’s B5 medium (half-strength Gamborg’s B5 (Merck, Darmstadt, Germany, G5768) with 2.5 mM MES-KOH (pH 5.7)) [24] supplemented with 1% (*w*/*v*) sucrose and 1% (*w*/*v*) phytoagar (Duchefa Biochemie, Haarlem, The Netherlands, P1003). Unless otherwise specified, the plants were grown under continuous white fluorescent light at an intensity of 50 µmol m^−2^ s^−1^ at 22 °C.

### 2.2. Analysis of Thallus Size and Regeneration

Marchantia gemmae were grown on ½ Gamborg’s B5 medium plates supplemented with 1% sucrose for 12 days, and thalli from three plants were then chopped into fragments smaller than 2 mm using a sterilized razor blade. This process generated approximately two thousand fragments. The fragments were washed four times with the growth medium. The fragments were spread onto ½ Gamborg’s B5 medium with 1% sucrose plates and incubated at 22 °C under continuous white, fluorescent light for 7 days. Images of 16 plate sections were captured using a stereomicroscope on days 0 and 7 to assess survival and growth. The survival rate was determined based on the presence of viable green tissue and the visible regeneration of new tissue. The size of the thallus fragments was measured using Fiji/ImageJ software (version 2.16.0; Fiji developers, 2024).

### 2.3. Plasmid Construction

The reporter gene *β-glucuronidase* (*uidA*/*GUS*) expressing construct (EF1pro:GUS) was generated as follows. The *GUS* gene was amplified with the primers GUS_fuF: 5′-AAGGAACCAATTCAGTCGACATGTTACGTCCTGTAGAAACCCC-3′ and GUS_fuR: 5′-AAGAAAGCTGGGTCTAGATATCATTGTTTGCCTCCCTGC-3′. The amplified fragment was cloned into the *Sal*I/*EcoR*V-digested pENTR1A vector (Thermo Fisher Scientific, Waltham, MA, USA) via Gibson assembly. The *GUS* gene was subsequently transferred into the pMpGWB303 plasmid [25] using Gateway LR cloning (Thermo Fisher Scientific). This construct enables the expression of GUS under the control of the Marchantia elongation factor 1-α (EF1α) promoter.

### 2.4. Bacterial Strain and Culturing

*Agrobacterium tumefaciens* strain EHA101 carrying the EF1pro:GUS plasmid was used in all experiments. The EH101 strain has high transformation efficiency in Marchantia transformation [7]. For transformation, *Agrobacterium* cultures were initiated from a single colony and grown in 5 mL of LB medium at 30 °C with overnight agitation. The bacterial culture (1 mL) was centrifuged to pellet the cells, which were then resuspened in 2.5 mL of ½ Gamborg’s B5 medium supplemented with 1% (*w*/*v*) sucrose and 100 µM 3,5-dimethoxy-4-hydroxyacetophenone (acetosyringone). The suspension was incubated at 30 °C for 3–6 h to induce virulence before co-cultivation with Marchantia thallus fragments. All procedures were performed aseptically.

### 2.5. Agrobacterium-Mediated Transformation of Marchantia

For the chopped-thallus method, a total of ten 12- to 14-day-old Marchantia plants were collected and placed in a plastic Petri dish with 3–5 mL of liquid medium. Using a sterilized razor blade, the thalli were finely chopped into fragments smaller than 2 mm within a laminar flow cabinet. The fragments were washed three times with a liquid medium using a cell strainer. The chopped thallus pieces were cultured in 50 mL of ½ Gamborg’s B5 medium with 1% sucrose or a supplemented medium containing 1% sucrose, 0.03% L-glutamine, and 0.1% of Casamino acid in a 250-mL flask capped with aluminum foil. Cultures were incubated under continuous white fluorescent light (50 µmol m^−2^ s^−1^) at 22 °C for 3–7 days with agitation at 180 rpm on a shaker to promote regeneration. Before co-cultivation, an additional 2.5 mL of 20% sucrose (equivalent to 1% sucrose in the total volume) was added to the flasks containing medium with 1% sucrose. While this brought the theoretical total sucrose concentration to 2%, the actual concentration may have been lower due to the consumption of sucrose by the thallus fragments during regeneration. One milliliter of *Agrobacterium* culture (prepared as described in Section 2.4) and 100 µM acetosyringone were then added. Co-cultivation was performed with agitation at 180 rpm at 22 °C in the dark for 3 days. Following co-cultivation, the fragments were washed with sterilized water four times. Then, the culture from a flask was transferred to a Falcon tube and allowed to settle briefly, enabling the fragments to sediment. The supernatant was carefully removed as much as possible, and sterilized water was added to the tube. The contents were shaken or vortexed, and the supernatant was removed as described above. Sterilized water was added again, and the contents were shaken or vortexed before being poured through a cell strainer. The fragments were subsequently rinsed twice with sterilized water. The washed fragments were distributed among three selection plates containing ½ Gamborg’s B5 medium supplemented with 0.5 µM chlorsulfuron and 100 mg/L cefotaxime. In each plate, 1–2 mL of sterile water was added, and the plate was tilted to ensure the water spread evenly over the surface before incubation under continuous white light at 22 °C.

For the traditional thallus method [6], ten 12- to 14-day-old Marchantia plants were harvested, and their apical meristems were removed. Each thallus was divided into two pieces, resulting in four pieces per plant. These fragments were cultured on a solid ½ Gamborg’s B5 medium with 1% sucrose and 1% agar under continuous light at 22 °C for 3 days to promote regeneration. Forty regenerating plantlets were co-cultivated with 1 mL of *Agrobacterium* culture (prepared as described in Section 2.4) in 50 mL of ½ Gamborg’s B5 medium containing 2% sucrose. Additionally, co-cultivation was performed with medium supplemented with 0.03% L-glutamine and 0.1% casamino acid in a 250-mL flask. Both cultures were supplemented with 100 µM acetosyringone. The subsequent washing and selection steps were identical to those used for the chopped-thallus method.

### 2.6. GUS Enzyme Activity Check via Histochemical Assay

Histochemical assays for GUS enzyme activity were conducted with slight modifications based on previously described methods [26]. Plant tissues were vacuum-infiltrated with GUS assay solution for 30 min and incubated for up to 2 h at 37 °C. The GUS assay solution contained 100 mM sodium phosphate buffer (pH 7.0), 0.5 mM potassium ferrocyanide, 0.5 mM potassium ferricyanide, 10 mM EDTA, 0.01% (*v*/*v*) Triton X-100, and 1 mM 5-bromo-4-chloro-3-indolyl-β-glucuronide (X-Glc). Ethanol was used to clear pigments, ensuring better visualization of GUS staining.

### 2.7. Genomic DNA Isolation and PCR Analysis

Genomic DNA was extracted from transformed Marchantia to confirm the integration of T-DNA. Approximately 50 mg of thallus tissue was homogenized in a microcentrifuge tube with 50 µL of DNA extraction buffer (200 mM Tris-HCL (pH 7.5), 250 mM NaCl, 25 mM EDTA, and 0.5% (*w*/*v*) SDS). The homogenized sample was mixed with 100 µL of absolute ethanol, vortexed, and centrifuged at maximum speed for 5 min at 22 °C. The resulting pellet was vacuum-dried and dissolved in Tris-EDTA buffer (pH 8.0). For PCR analysis, the extracted genomic DNA was amplified using the primers GUS_FP (5′-ATACCGAAAGGTTGGGCAGG-3′) and GUS_RP (5′-TCTTGCCGTTTTCGTCGGTA-3′) to detect the integration of the GUS transgene. Y chromosome-specific region rhm12 [27] was amplified as a positive control using primers: rhm12_for (5′-GAGAGTATTTGCGATGCGTCAC-3′) and rhm12_rev (5′-CAAGGGCTCGAATCCATTTCT-3′).

### 2.8. Quantitative Real-Time PCR (qPCR)

Total RNA was isolated from the 1 cm tip of the thallus of Marchantia plants using TRIzol (Thermo Fisher Scientific, 15596018). First-strand cDNA was synthesized using Ready-to-Go RT-PCR beads (Cytiva, Marlborough, MA, USA, 27925901) with random hexamers. qPCR analyses were performed using the QuantStudio 6 (Thermo Fisher Scientific) and PowerUp SYBR Green Master Mix (Thermo Fisher Scientific, A25742) according to the manufacturer’s instructions. *MpACT* was used as an internal control, with primers 5′-AGGCATCTGGTATCCACGAG-3′ and 5′-ACATGGTCGTTCCTCCAGAC-3′ [28]. The GUS gene was quantified using the previously mentioned GUS_FP and GUS_RP primers. Quantification was performed using a standard curve.

### 2.9. Statistical Analysis

Each transformation test was validated through three independent experiments, using 9–11 individual plants in each test. Statistical analysis was conducted using R software (version 3.2.1; R Core Team, 2025). Data were analyzed using one-way ANOVA followed by Tukey’s HSD test at a 0.05 significance level.

## 3. Results

### 3.1. Thallus Finely Chopped into Micro-Fragments Generates a Large Number of Regenerated Plants

As previously reported, excised pieces of thallus produce visible tissue five days after plating on the growth medium, suggesting that regenerating thalli undergo extensive cell division without the application of growth regulators [6,29]. Fragments traditionally used in thallus transformation are approximately 5–8 mm in size. This study aimed to determine whether smaller thallus fragments could regenerate effectively.

**Figure 1:**
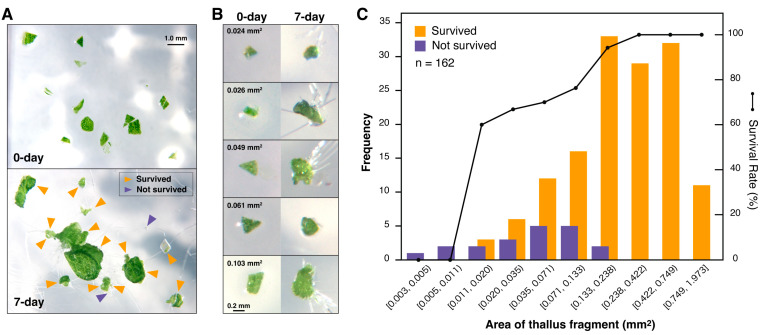
Survival rate of chopped thalli. (**A**) Thalli from 12-day-old plants were finely chopped into fragments smaller than 2 mm using a razor blade. These fragments were incubated on growth medium plates at 22 °C under continuous light for 7 days, and their growth was observed under a microscope. Survival was determined by green coloration and visible cell proliferation. Survived and not survived are indicated by orange and purple arrowheads, respectively. (**B**) Magnified images of surviving thallus fragments. The numbers in the top-left corner represent the area of each fragment. (**C**) A histogram showing the number of surviving and dead thallus fragments based on their size. The survival rate for each size category is represented as a line graph derived from this data. The data were binned using logarithmic scaling. A total of 162 thallus fragments were analyzed.

To investigate this, thalli, which have developed from gemmae and 12-day-old plants with dichotomous branches and meristems at each growth tip, were chopped into fragments smaller than 2 mm using a sterilized razor blade (Figure 1A). This procedure generated approximately 700 or more fragmented thalli per plant. The fragments were placed on a solid growth medium and incubated under standard growth conditions. The fragmented thalli exhibited substantial regenerative capacity, with 88% of the fragments surviving. Even fragments as small as 0.2 mm regenerated and formed new tissues (Figure 1B). Survival rates varied with fragment size: fragments measuring 0.19–0.27 mm (0.035–0.071 mm^2^) achieved over 70% survival, while fragments larger than 0.49 mm consistently demonstrated 100% survival and regeneration (Figure 1C).

This study demonstrates that the chopped thallus fragments can regenerate, highlighting the robustness of Marchantia in tissue regeneration. Several factors may contribute to the lower survival rate of smaller fragments. These include increased physical damage due to a higher proportion of cut surfaces and a reduced percentage of cells in a regeneration-compatible state. However, since chopping produces hundreds of fragments per plant and 88% of these fragments survive, this issue is unlikely to have a significant impact. Larger fragments are more likely to contain intact and viable cells, which may explain their higher regeneration rates. Nonetheless, it is worth emphasizing that most fragmented thallus pieces, except for the extremely small ones, regenerated successfully.

### 3.2. Establishing the Chopped-Thallus Method for Thallus Transformation

In spore-based Marchantia transformation methods, immature thalli derived from spores are typically used [5,10]. The chopped-thallus method proposed in this study generates fragments smaller than those traditionally used in thallus-based transformations, yet they remain suitable for genetic modification. To evaluate the transformation efficiency of this method, we followed the protocol described by Kubota et al., 2013 [6]. Hereafter, we refer to the chopped-thallus approach as the “chopped-thallus method” and the established approach as the “traditional method”.

**Figure 2:**
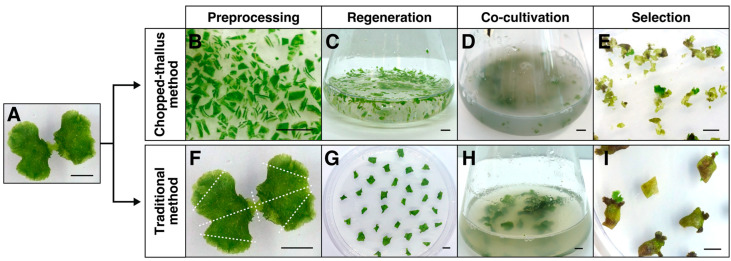
Workflow of thallus transformation. Marchantia plants aged 12–14 days (**A**) were used as materials for transformation using either the chopped-thallus method (**B**–**E**) or the traditional method (**F**–**I**). Bars = 5 mm.

Thalli grown on a solid medium for 12–14 days from gemmae served as the starting material for transformation (Figure 2A). For the chop-thallus method, the thalli were finely chopped into fragments (<2 mm) using a razor blade (Figure 2B). Regeneration of these fragments was performed in liquid ½ Gamborg’s B5 medium supplemented with 1% sucrose (Figure 2C), allowing co-cultivation with *Agrobacterium* to proceed in the same container, eliminating the need to transfer the fragments. The fragments were co-cultivated with *Agrobacterium* carrying reporter genes (GUS and chlorsulfuron-resistant gene) (Figure 2D). Sucrose is essential for successful transformation [6]. In this study, 1% sucrose was added to the co-cultivation medium, resulting in a total of 2% sucrose during co-cultivation, accounting for the sucrose already present. Acetosyringone was also added to enhance *Agrobacterium* virulence. In the traditional method, plants were processed by removing apical meristems and dividing the thalli into four pieces, followed by regeneration on a solid growth medium (Figure 2F,G). These regenerated fragments were co-cultivated with *Agrobacterium* (Figure 2H).

**Figure 3:**
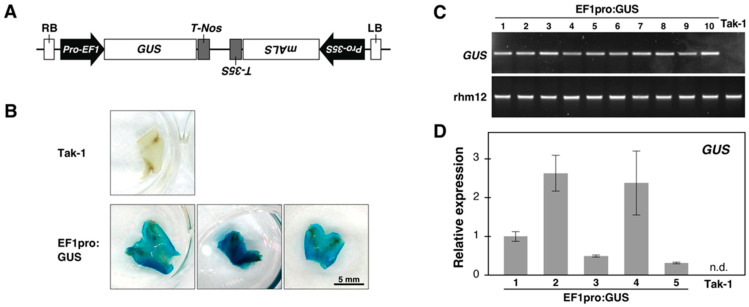
Confirmation of transformation. (**A**) Schematic representation of the EF1pro:GUS construct. The probe region indicates the primer binding site for the GUS gene. GUS—β-glucuronidase; mALS—mutated acetolactate synthase (chlorsulfuron resistance gene); Pro-EF1—promoter of Marchantia elongation factor 1-α; Pro-35S—cauliflower mosaic virus 35S promoter; T-Nos—nopaline synthase terminator; T-35S—cauliflower mosaic virus 35S terminator; RB—Right border; LB—Left border. (**B**) Transgene expression was confirmed through GUS staining. The plants were grown for three weeks on the selection medium. (**C**) PCR analysis was performed on genomic DNA extracted from randomly selected chlorsulfuron-resistant plants. (**D**) Stable transgene expression in the thallus tips of 6-week-old plants was confirmed by qPCR. Bars represent the mean of three biological replicates with standard error. n.d.—not detected.

After chlorsulfuron selection (Figure 2E,I), the integration of transgenes was confirmed through histochemical GUS staining, PCR analysis of genomic DNA, and the verification of stable transgene expression by qPCR (Figure 3).

### 3.3. The Chopped-Thallus Method Simplifies Transformation and Enhances Efficiency

Kubota et al. (2013) [6] optimized the *Agrobacterium*-mediated thallus transformation protocol and established a consistent and reproducible method. Their key findings included: (i) a 3-day regeneration period was sufficient for effective transformation, (ii) the presence of sucrose during co-cultivation significantly enhanced transformation efficiency, and (iii) the highest transformation efficiency was achieved using the M51C medium rather than ½ Gamborg’s B5 medium. To develop the chopped-thallus method, we adopted two critical factors: (i) a 3-day regeneration period and (ii) the addition of sucrose to the co-cultivation medium. Regarding light conditions, a previous report on the AgarTrap method investigated light conditions and found higher transformation efficiency in the dark [7]. Based on this, co-cultivation was also performed in the dark in our study. Instead of the labor-intensive M51C medium [17], we used the simpler and more readily available ½ Gamborg’s B5 medium, leveraging the high material output generated by the chopped-thallus method.

**Figure 4:**
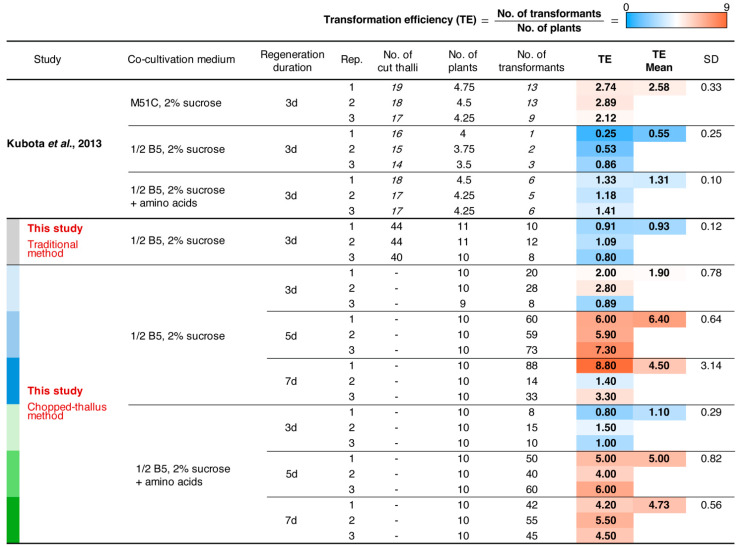
Comparison of transformation efficiency under different transformation conditions. The table summarizes the transformation efficiency for various co-cultivation media, regeneration durations, and methods (traditional and chopped-thallus). The numbers shown in italics were taken from experiments reported by Kubota et al., 2013 [6] and were used to calculate the number of plants and transformation efficiency. Experiments conducted in this study were repeated three times (Rep.). Transformation efficiency (TE) was calculated as the number of transformants per plant. The mean TE and standard deviation (SD) are shown for each condition. A heat map is applied to the TE column. The color codes on the left correspond to the charts shown in Figure 5 and Figure 6.

**Figure 5:**
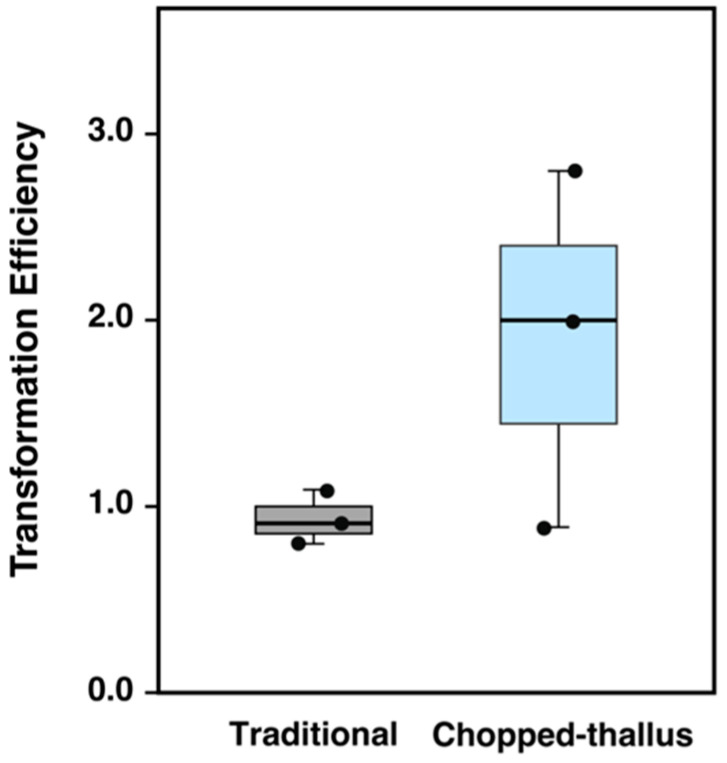
Comparison of transformation efficiency between the traditional and chopped-thallus methods. Plant fragments prepared by each method were regenerated for 3 days and then subjected to transformation. Co-cultivation was performed within ½ Gamborg’s B5 medium containing 2% sucrose. Transformation efficiency indicates the number of transformants per plant. Data represent the results of three independent experiments.

We compared the transformation efficiencies of the chopped-thallus method and the traditional methods using a ½ Gamborg’s B5 medium with a 3-day regeneration period. Although the difference was not statistically significant, a clear trend indicated that the chopped-thallus method nearly doubled the transformation efficiency compared to the traditional method (Figure 4 and Figure 5). The lower efficiency observed in the traditional method with ½ Gamborg’s B5 medium aligns with previous findings [6]. These results indicate that chopping increases the amount of transformation-compatible material, thus mitigating the limitations associated with using ½ Gamborg’s B5 medium.

### 3.4. Optimizing Regeneration Period and Medium Composition

We further evaluated the optimal regeneration period for chopped thalli over 3–7 days, as finely chopped thalli might sustain more damage than those used in the traditional methods. Simultaneously, we investigated the effect of supplementing the co-cultivation medium with amino acids (L-glutamine and Casamino acid), which have been reported to enhance transformation efficiency [6].

**Figure 6:**
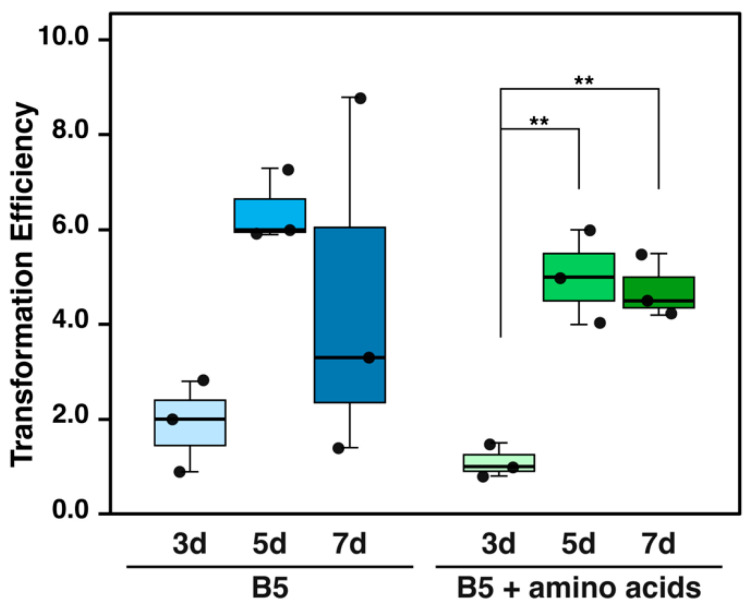
Comparison of transformation efficiency between different regeneration periods and co-cultivation media in chopped-thallus method. Regeneration periods of 3, 5, and 7 days were assessed. Two types of co-cultivation media were examined: ½ Gamborg’s B5 medium containing 2% sucrose (B5) and B5 supplemented with 0.03% L-glutamine and 0.1% Casamino acid (B5 + amino acids). Transformation efficiency indicates the number of transformants per plant. Data represent the results of three independent experiments. Statistical analysis was performed separately for each medium group using one-way ANOVA followed by Tukey’s HSD test. ** *p* < 0.01.

Extending the regeneration period from 3 to 5 days significantly improved transformation efficiency. This trend was observed in both ½ Gamborg B5 medium and the medium supplemented with amino acids (Figure 4 and Figure 6). In the amino acids-supplemented medium, the transformation efficiency for the 5-day regeneration period (5.00 ± 0.82) was significantly higher than for 3 days (1.10 ± 0.29). In ½ Gamborg’s B5 medium without supplementation, the transformation efficiency for 5 days (6.40 ± 0.64) showed no significant difference compared to 3 days (1.90 ± 0.78), although a potential improvement was noted.

Notably, the 5-day regeneration period achieved approximately 2.5 times higher transformation efficiency than the traditional method using M51C medium (2.48 ± 0.33) (Figure 4). However, no additional benefits were observed with a 7-day regeneration period compared to 5 days. Additionally, excessive growth of the thallus fragments during the 7-day period made the procedure less practical. Therefore, a regeneration period of 4–5 days was determined to be optimal for the chopped thallus method. The supplementation of amino acids did not have a consistent effect on transformation efficiency, suggesting that ½ Gamborg’s B5 medium alone is sufficient for this method.

## 4. Discussion

**Figure 7:**
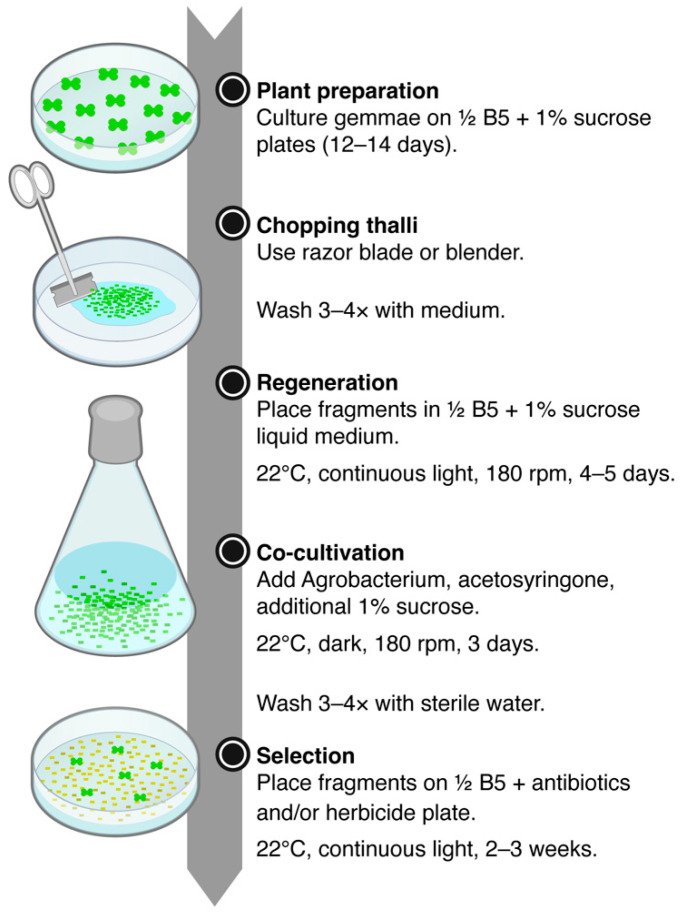
Workflow of the chopped-thallus transformation method.

Figure 7 illustrates one of the recommended workflows of the chopped-thallus transformation method. While this study primarily used a razor blade, using an electric blender instead can make the process more efficient. In addition, gemmae can be cultured on sterilized cellophane sheets placed over agar plates, allowing the plants to be easily peeled off and collected for chopping.

Table 1 compares the chopped-thallus method with other standard methods. The Traditional method involves removing the apical meristems from the four growth tips of the thallus, quartering the thallus, and placing the pieces onto a fresh growth medium. Processing a small number of plants does not take much time; handling 10 plants takes approximately 5–10 min. When using the M51C medium, this method yields approximately 26 transformants from ten plants [6] (or 10 transformants with Gamborg’s B5 medium, Figure 5). If this number of transformants is sufficient for an experiment, the traditional method may be the preferred choice. In the chopped-thallus method, all plants are fragmented at once. The chopping step itself takes around two minutes, but additional steps are required, including washing the fragments 3–4 times using a cell strainer and transferring them to liquid culture vessels such as flasks or beakers. In total, the process takes approximately 10 min. Regarding resource use, the chopped-thallus method is more efficient than other methods. With Gamborg’s B5 medium, this method yields around 64 transformants from 10 plants. However, such a high number of transformants may not always be required depending on the experiment. Thus, the choice of method should depend on the experimental design and scale.

In addition to the traditional thallus transformation method, the AgarTrap method is also an efficient transformation approach. This method features a straightforward workflow that combines plant culturing, Agrobacterium co-cultivation, washing, and selection on a single plate without requiring a shaker. This allows for the continuous use of the same medium throughout the process [7,10,30]. When using gemmae, the transformation efficiency is relatively high; nearly 100% of gemmae produce transformants (Table 1) [7]. However, concerns include the occasional overgrowth of *Agrobacterium*, which makes continuous transformation complicated, and the fact that AgarTrap is not compatible with gentamicin selection [7], making it less convenient for experiments requiring multiple constructs. For large-scale experiments, another issue is the washing step. The *Agrobacterium* growing on the medium must be removed by pipetting, which is time-consuming and requires careful handling. Due to these factors, the AgarTrap method may not be ideal for large-scale applications.

Based on our experience, transformation efficiency can vary depending on the type of construct used. Simple constructs, such as those that only include fluorescent markers, typically perform well without any issues. In contrast, more complex constructs, which may contain large genes or multiple inserts, sometimes demonstrate lower transformation efficiency. Supplementary Figure S1 shows an example where transformation with a certain construct initially failed using the AgarTrap method but was successfully achieved with the chopped-thallus method. While it remains to be determined whether this success was due to the higher transformation efficiency of the chopped-thallus method or because the AgarTrap method was not suitable for this particular construct, having multiple method options provides greater flexibility in addressing such challenges. Furthermore, the chopped-thallus method is particularly effective for large-scale mutant screening, where a high number of transformants is needed. This is especially valuable for lines that cannot produce spores, making it a powerful tool for transformation in such backgrounds.

We investigated whether Gamborg’s B5 medium could provide sufficient transformation efficiency in the chopped-thallus method. M51C medium requires mixing more than ten different reagents and microelements [17], whereas Gamborg’s B5 medium is widely used globally and commercially available from various suppliers. While there may be a trade-off between convenience and transformation efficiency in this method, an overall improvement in transformation efficiency has been achieved. So far, we have not tested other common media, such as Murashige and Skoog medium, but it would be worth testing in the future. In recent studies on hornwort transformation, the optimal pH for co-cultivation was reported to be 5.0–5.5 (compared to 5.7 in this study) [22]. Since pH optimization for transformation in Marchantia has not been explored yet, it would be valuable to investigate this. Furthermore, optimizing the timing of chopping could improve efficiency; however, these challenges remain for future studies.

## 5. Conclusions

The primary objective of this study was to enhance transformation efficiency and simplify the methodology by developing a modified chopped-thallus method. This method uses the entire plant, significantly reducing processing time and maximizing the use of limited plant resources. The chopped-thallus method demonstrated that ½ Gamborg’s B5 medium can effectively substitute for M51C, simplifying the protocol without compromising transformation efficiency. Additionally, supplementation of amino acids to the co-cultivation medium did not provide any significant benefit. The optimal regeneration period for fragmented thalli was estimated to be 4–5 days.

The proposed method is particularly suitable for large-scale transformations, as it eliminates the need to remove meristems or divide each thallus manually, allowing the preparation of numerous thallus fragments simultaneously. This approach facilitates high-throughput experiments, including mutant screening, by enabling the efficient handling of numerous samples. This simplified and scalable method has the potential to advance genetic studies and support large-scale applications in Marchantia research and biotechnology.

## Figures and Tables

**Table 1:** Comparison of Marchantia transformation methods.

			Media and Durations ^1,2^								
	Study	Starting Material	Plant Development (Duration)	Pre-Culture/ Regeneration (Duration)	Co-Culture (Duration)	Selection	Transformation Efficiency ^3^	Estimated Number of Transformants per Experiment	Uniform Genetic Background	Advantage	Disadvantage
Traditional	Ishizaki et al. 2008 [5]	Spore	-	M51C ^4^ liquid (5 d)	------> (7 d)	M51C ^4^ solid	N.D. ^5^ (600–800 transformants per sporangium)	-	no	-	-
	Kubota et al. 2013 [6]	Thallus	1/2 B5 solid (2 w)	1/2 B5 + sucrose solid (3 d)	M51C ^4^ liquid (3 d)	1/2 B5 solid	2.58 ± 0.33	26 transformants (when 10 plants are used with 1 flask and 1 selection plate)	yes	One of the most common and reliable methods; low residual *Agrobacterium*	Meristem removal and quartering; transferring at each stage; achieving high transformation efficiency requires M51C medium; requires a shaker.
AgarTrap	Tsuboyama et al. 2014 [10]	Spore	-	M51C ^4^ solid (3 d)	------> (3 d)	------>	0.16 ± 0.09	-	no	-	-
	Tsuboyama-Tanaka et al. 2015 [30]	Thallus	1/2 B5 solid (1 m)	M51C ^4^ solid (0 d)	------> (3 d)	------>	0.55 ± 0.05 ^6^	-	yes	-	-
	Tsuboyama et al. 2018 [7]	Gemma	-	1/2 B5 + sucrose solid (2 d)	------> (2 d)	------>	0.97 ± 0.05 ^7^	49 transformants (when 50 gemmae in 1 plate are used)	yes	All steps completed on a B5 plate; shorter duration due to the use of gemmae.	*Agrobacterium* overgrowth; affected by humidity conditions; incompatible with gentamycin selection.
Chopped-thallus	This study	Thallus	1/2 B5 + sucrose solid (2 w)	1/2 B5 + sucrose liquid (4 d)	------> (3 d)	1/2 B5 solid	6.40 ± 0.64	64 transformants (when 10 plants are used with 1 flask and 3 selection plates)	yes	A single plant generates hundreds of fragments; no precise dissection required; scalable; B5 medium is sufficient; low residual *Agrobacterium*.	Requires a longer duration and additional chopping effort compared to the AgarTrap method; requires a shaker.

^1^ Duration: d, w, and m indicate days, weeks, and months, respectively. ^2^ Arrows (------>) indicate the continuation of the same medium without transfer. ^3^ Transformation efficiency was calculated as the number of transformants per number of plants used. ^4^ M51C medium contains sucrose [17]. ^5^ N.D.—Not Determined. ^6^ With male thalli. ^7^ Using EHA101 *Agrobacterium* under dark conditions.

## Data Availability

Data on the area and regeneration rate of Marchantia thallus fragments are available on the Zenodo repository: https://doi.org/10.5281/zenodo.14655757. Other data sets are available upon request from the authors.

## Supplementary Materials

The following supporting information can be downloaded at: https://www.mdpi.com/article/10.3390/plants14040582/s1, Figure S1: Comparison of transformations using the problematic plasmid. The pMpGE010 plasmid [31] harboring *ATG9* gRNA (*atg9-2*) and the *Cas9* gene in EHA101 *Agrobacterium* was used to transform the host plant Tak-1 (which expresses the peroxisome marker Citrine-PTS1) via the G-AgarTrap and chopped-thallus methods. For the chopped-thallus method, one of the four plates used for selection is shown. Selection was carried out using the hygromycin resistance marker for three weeks. The table shows the amount of materials used and the number of transformants obtained in six AgarTrap experiments and one chopped-thallus experiment.

## Abbreviations

The following abbreviations are used in this manuscript:

ANOVA	Analysis of variance
EF1	Elongation factor 1-α
GUS	β-glucuronidase
HSD	Honestly significant difference
LB	Left border
mALS	mutated acetolactate synthase
Marchantia	*Marchantia polymorpha*
RB	Right border
Tak	Takaragaike
TE	Transformation efficiency
