# Supplementary material for: A Simple and Scalable Chopped-Thallus Transformation Method for Marchantia polymorpha
Source: Plants (Basel). 2025 Feb 14;14(4):582. doi: 10.3390/plants14040582 (PMC11859832; doi:10.3390/plants14040582)
Supplement: Supplementary file 1 [file plants-14-00582-s001.zip › plants-3459625-supplementary.pdf]

Supplementary Information

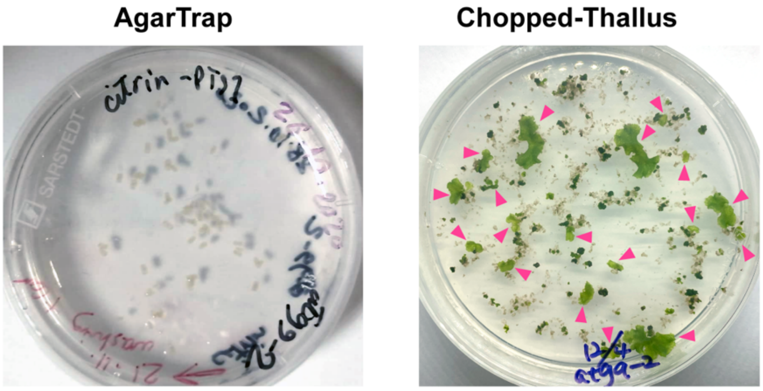

	Experiment	Material	Number of transformants
G-AgarTrap	# 1	approx. 50 gemmae	0
	# 2	approx. 50 gemmae	0
	# 3	approx. 50 gemmae	0
	# 4	approx. 50 gemmae	0
	# 5	approx. 50 gemmae	0
	# 6	approx. 50 gemmae	0
Chopped-thallus	# 1	18 plants	36

Supplementary Figure S1. Comparison of transformations using the problematic plasmid. The pMpGE010 plasmid [1] harboring *ATG9* gRNA (*atg9-2*) and the *Cas9* gene in EHA101 *Agrobacterium* was used to transform the host plant Tak-1 (which expresses the peroxisome marker Citrine-PTS1) via the G-AgarTrap and chopped-thallus methods. For the chopped-thallus method, one of the four plates used for selection is shown. Selection was carried out using the hygromycin resistance marker for three weeks. The table shows the amount of materials used and the number of transformants obtained in six AgarTrap experiments and one chopped-thallus experiment.

Reference

1. Sugano, S. S.; Nishihama, R.; Shirakawa, M.; Takagi, J.; Matsuda, Y.; Ishida, S.; Shimada, T.; Hara-Nishimura, I.; Osakabe, K.; Kohchi, T. Efficient CRISPR/Cas9-based genome editing and its application to conditional genetic analysis in *Marchantia polymorpha*. *PLoS One* **2018**, *13* (10), e0205117. DOI: 10.1371/journal.pone.0205117.
